# First Identified Cases of SARS-CoV-2 Variant B.1.1.7 in Minnesota — December 2020–January 2021

**DOI:** 10.15585/mmwr.mm7008e1

**Published:** 2021-02-26

**Authors:** Melanie J. Firestone, Alexandra J. Lorentz, Xiong Wang, Kathryn Como-Sabetti, Sara Vetter, Kirk Smith, Stacy Holzbauer, Stephanie Meyer, Kristin Ehresmann, Richard Danila, Ruth Lynfield

**Affiliations:** ^1^Minnesota Department of Health; ^2^Epidemic Intelligence Service, CDC; ^3^Division of State and Local Readiness, Center for Preparedness and Response, CDC.

On January 9, 2021, the Minnesota Department of Health (MDH) announced the identification of the SARS-CoV-2 variant of concern (VOC) B.1.1.7, also referred to as 20I/501Y.V1 and VOC 202012/01, in specimens from five persons; on January 25, MDH announced the identification of this variant in specimens from three additional persons. The B.1.1.7 variant, which is reported to be more transmissible than certain other SARS-CoV-2 lineages*^,^^†^ (*1*), was first reported in the United Kingdom in December 2020 (*1*). As of February 14, 2021, a total of 1,173 COVID-19 cases of the B.1.1.7 variant had been identified in 39 U.S. states and the District of Columbia (*2*). Modeling data suggest that B.1.1.7 could become the predominant variant in the United States in March 2021 (*3*).

The B.1.1.7 variant has a mutation in the spike protein that causes S-gene target failure (SGTF) in the Thermo Fisher Scientific TaqPath COVID-19 reverse transcription–polymerase chain reaction (RT-PCR) assay. The overall RT-PCR result is positive but is negative for the S-gene target and positive for the other two assay targets; SGTF has served as a proxy for identifying the B.1.1.7 variant (*1*). The MDH Public Health Laboratory (MDH-PHL) requested SARS-CoV-2 RT-PCR–positive specimens with SGTFs collected during November 1, 2020–January 12, 2021, from clinical laboratories that used the TaqPath assay, and 30 specimens were received. An additional specimen that had been collected from a household contact of a person with an SGTF specimen was requested and obtained from a clinical laboratory using another COVID-19 assay that does not detect SGTFs. MDH-PHL conducted whole genome sequencing to analyze the 31 specimens.^§^

The SARS-CoV-2 variant B.1.1.7 was identified in eight specimens from Minnesota residents, including six (19%) of the 31 specimens sequenced by MDH-PHL and two specimens sequenced through CDC’s national SARS-CoV-2 surveillance system.^¶^ The eight specimens were collected during December 18, 2020–January 11, 2021, from eight Minnesota residents in five counties in the Minneapolis–St. Paul metropolitan area. Seven persons were interviewed after receiving positive SARS-CoV-2 test results; after those with the B.1.1.7 variant were identified, MDH case investigators recontacted the patients to obtain additional information on exposures and close contacts. Six of the eight patients were successfully contacted, including one who had not been interviewed previously. This activity was reviewed by CDC and was conducted consistent with applicable federal law and policy.^**^

The eight persons from whom the specimens were collected ranged in age from 15 to 41 years. Three persons had a history of international travel during the 14 days before illness onset, including two who traveled to West Africa (MN-MDH-2252 and MN-MDH-2254) (Figure) and one who traveled to the Dominican Republic (MN-CDC-STM-0000013). Three additional persons traveled to California (MN-MDH-2415, MN-MDH-2416, and MN-CDC-STM-153) in the 14 days before illness onset or specimen collection, including one who received a positive test result while in California and isolated there before returning to Minnesota. Five persons reported COVID-19–like symptoms and had illness onset dates during December 16, 2020–January 10, 2021; three were asymptomatic. Two sequences (MN-MDH-2253 and MN-MDH-2255) were identical, and the MN-MDH-2252 sequence differed by one single nucleotide variant (SNV). The three sequences for cases from California clustered together within one to three SNVs and are genetically distinct from the other sequences. Two specimens from international travelers, MN-MDH-2254 and MN-CDC-STM-0000013, did not have sequences similar to those identified in Minnesota.

**FIGURE:**
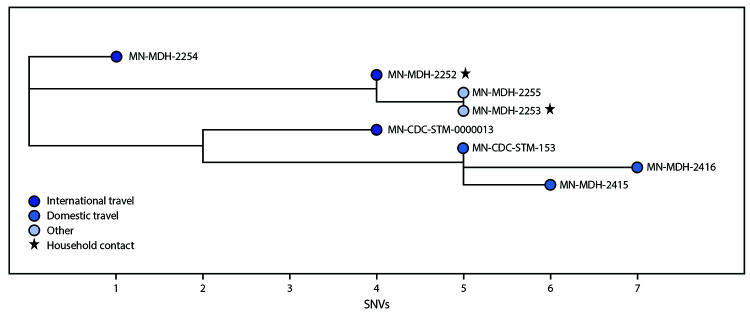
Phylogenetic tree* showing genetic distance^†^ between SARS-CoV-2–positive specimens with the B.1.1.7 variant (n = 8) and exposure histories related to travel,^§^ household contacts, and others in the community **Abbreviation**: SNV = single nucleotide variant. * Phylogenetic tree created using Interactive Tree of Life (version 5.7; European Molecular Biology Laboratory). https://itol.embl.de/ ^†^ MN-MDH-2253 and MN-MDH-2255 were identical, and MN-MDH-2252 was within one SNV. MN-MDH-2252 and MN-MDH-2253 were collected from persons who were household contacts. Two specimens from international travelers, MN-MDH-2254 and MN-CDC-STM-0000013, did not have sequences similar to those identified in Minnesota. ^§^ International and domestic travel occurred during the 14 days before illness onset or specimen collection, including onset of illness in persons who received positive SARS-CoV-2 test results while away from Minnesota. MN-CDC-STM-153, MN-MDH-2416, and MN-MDH-2415 were clustered together within one to three SNVs. These three specimens were from persons who reported travel to California in the 14 days before illness onset or specimen collection, including one who received a positive test result while in California and isolated there before returning to Minnesota.

Persons identified with the variant B.1.1.7 in Minnesota had exposure histories related to travel (six), the household (one), and others in the community (one). None had a history of travel to the United Kingdom, although three persons traveled internationally and three persons traveled domestically in the 14 days before illness onset or specimen collection, including one who received a positive test result before returning to Minnesota. Identification of this variant in Minnesota, a variant that epidemiologic and genomic evidence suggests has increased transmissibility, highlights the importance of mitigation measures such as mask use, physical distancing, avoiding crowds and poorly ventilated indoor spaces, isolation of persons with diagnosed COVID-19, quarantine of close contacts of persons with COVID-19,^††^ and adherence to CDC travel guidance^§§^ to slow transmission. As SARS-CoV-2 continues to evolve, timely genomic surveillance and disease mitigation strategies will be critical for monitoring variant emergence and protecting public health.

## Footnotes

*
https://www.medrxiv.org/content/10.1101/2020.12.30.20249034v1.full.pdf

†
https://www.biorxiv.org/content/10.1101/2020.12.14.422555v3.full.pdf

§
https://virological.org/t/tracking-sars-cov-2-voc-202012-01-lineage-b-1-1-7-dissemination-in-portugal-insights-from-nationwide-rt-pcr-spike-gene-drop-out-data/600

¶
https://www.cdc.gov/coronavirus/2019-ncov/cases-updates/variant-surveillance.html

** 45 C.F.R. part 46, 21 C.F.R. part 56; 42 U.S.C. Sect. 241(d); 5 U.S.C. Sect. 552a; 44 U.S.C. Sect. 3501 et seq.

††
https://www.cdc.gov/coronavirus/2019-ncov/your-health/need-to-know.html

§§
https://www.cdc.gov/coronavirus/2019-ncov/travelers/travel-during-covid19.html
